# Analytic Methods for Understanding the Temporal Patterning of Dietary and 24-H Movement Behaviors: A Scoping Review

**DOI:** 10.1016/j.advnut.2024.100275

**Published:** 2024-07-18

**Authors:** Rebecca M Leech, Stephanie E Chappel, Nicola D Ridgers, Heather A Eicher-Miller, Ralph Maddison, Sarah A McNaughton

**Affiliations:** 1 Institute for Physical Activity and Nutrition, Deakin University, Geelong, Victoria, Australia; 2 Central Queensland University, Appleton Institute, School of Health, Medical and Applied Sciences, Adelaide, South Australia, Australia; 3 Alliance for Research in Exercise, Nutrition and Activity (ARENA), Allied Health and Human Performance, University of South Australia, Adelaide, South Australia, Australia; 4 Department of Nutrition Science, Purdue University, West Lafayette, IN, United States; 5 Health and Well-Being Center for Research Innovation, School of Human Movement and Nutrition Sciences, University of Queensland, St Lucia, Queensland, Australia

**Keywords:** temporal patterns, lifestyle behaviors, physical activity, sedentary behavior, diet, statistical methods, epidemiologic studies

## Abstract

Dietary and movement behaviors [physical activity (PA), sedentary behavior (SED), and sleep] occur throughout a 24-h day and involve multiple contexts. Understanding the temporal patterning of these 24-h behaviors and their contextual determinants is key to determining their combined effect on health. A scoping review was conducted to identify novel analytic methods for determining temporal behavior patterns and their contextual correlates. We searched Embase, ProQuest, and EBSCOhost databases in July 2022 to identify studies published between 1997 and 2022 on temporal patterns and their contextual correlates (e.g., locational, social, environmental, personal). We included 14 studies after title and abstract (*n* = 33,292) and full-text (*n* = 135) screening, of which 11 were published after 2018. Most studies (*n* = 4 in adults; *n* = 5 in children and adolescents), examined waking behavior patterns (i.e., both PA and SED) of which 3 also included sleep and 6 included contextual correlates. PA and diet were examined together in only 1 study of adults. Contextual correlates of dietary, PA, and sleep temporal behavior patterns were also examined. Machine learning with various clustering algorithms and model-based clustering techniques were most used to determine 24-h temporal behavior patterns. Although the included studies used a diverse range of methods, behavioral variables, and assessment periods, results showed that temporal patterns characterized by high SED and low PA were linked to poorer health outcomes, than those with low SED and high PA. This review identified temporal behavior patterns, and their contextual correlates, which were associated with adiposity and cardiometabolic disease risk, suggesting these methods hold promise for the discovery of holistic lifestyle exposures important to health. Standardized reporting of methods and patterns and multidisciplinary collaboration among nutrition, PA, and sleep researchers; statisticians; and computer scientists were identified as key pathways to advance future research on temporal behavior patterns in relation to health.

**Statement of Significance**

To our knowledge, this is the first comprehensive scoping review to identify novel methods for understanding the temporal patterning of dietary and 24-h movement behaviors over time and their contextual correlates. The identified temporal patterns and their reported associations with health outcomes suggest these methods hold promise for the discovery of holistic lifestyle exposures important to health.

## Introduction

Worldwide, the prevalence of suboptimal diet and insufficient physical activity (PA) remains unacceptably high [1,2]. Sedentary behavior (SED) and insufficient sleep also characterize modern lifestyles and may further contribute to ill health [3,4]. Adoption of healthy behaviors, including evidence-based recommendations for dietary and 24-h movement behaviors, which includes waking (i.e., PA and SED) and sleep behaviors, would confer substantial health and economic benefits such as a reduction in the global burden of noncommunicable diseases, lower health care costs, and higher workforce productivity [1,[3], [4], [5], [6]].

Dietary and 24-h movement behaviors occur at different times, frequencies, and sequences over time, herein referred to as temporal behavior patterns [[7], [8], [9]]. Throughout a 24-h day, people consume a variety of foods at eating occasions (i.e., meals and snacks), accumulate their time in daily PA and SED by moving at varying intensities, and go to sleep and wake up. Across the life course, levels of these same behaviors may also fluctuate and change, especially during periods of major life transitions [[10], [11], [12], [13], [14]], and the health effects of long-term exposure to unhealthy lifestyles are well documented [[15], [16], [17], [18]]. However, within a 24-h day, the accumulation in the timing, frequency, and sequencing of dietary and movement behaviors may have implications on health via the body’s circadian system. The body’s central clock governs circadian rhythms (24-h cycles) and works with peripheral clocks located in body organs to modulate physiologic and metabolic processes [19,20], which are responsive to external cues including light/dark exposure, PA, and eating/fasting [19]. Circadian misalignment of these cues impairs glycemic control, insulin responses, and blood pressure, and increasing evidence suggests that shift work, night eating, chronic social jet lag, sleep deprivation, and, conversely, oversleeping increase risk of obesity and cardiometabolic disorders [19,20].

Clustering describes how dietary, 24-h movement, and other behaviors occur in tandem and is a growing area of research [[21], [22], [23], [24], [25], [26], [27]]. Clustering captures population differences in behavior pattern combinations and interactions that may be obscured if levels of behaviors for a population are examined with disregard of individual differences [28,29]. For example, among youth, dietary and 24-h movement behaviors cluster in healthy, unhealthy, and mixed ways [22,23,25]. Although mixed patterns (e.g., high PA and high SED) are most prevalent [23,25], unhealthy patterns (e.g., high SED, high intake of energy-dense foods) have been linked to higher adiposity [23,25]. To date, these cluster patterns have mostly been based on daily/weekly averages of behaviors; temporal data have rarely been incorporated into patterns. An understanding of the when, frequency, duration, and type of behaviors may inform the design of precision health interventions. For example, some people may eat 6 times a day, consume most of their energy intake after 17:00 and engage in prolonged SED in the evening, whereas others may have evenly distributed energy intake across 3 mealtimes, be more physically active in the evening, and sleep 7 h a day. The temporal sequencing of behavior state transitions (i.e., going from active to sedentary, fasting to eating, awake to sleep) and behavior modalities [e.g., reading compared with watching television (TV), quality of sleep] also characterize daily temporal patterns and could be considered in the design of precision health interventions [30].

Dietary and 24-h movement behaviors are also influenced by a broad range of intrapersonal, social, cultural, economic, and political contextual factors that vary over time [[31], [32], [33]]. Examples include location, social setting, political/organizational setting, mood, motivational level, and concurrent activities (e.g., running while listening to music, eating while watching TV). Further, contextual factors may interact with dietary and 24-h movement behaviors to shape health. For example, emerging research suggests that people who undertake PA in greenspaces or share more meals with family have a lower risk of poor mental health than those who do not [34,35]. Understanding the accumulation of dietary and movement behaviors over time and their contextual determinants is key to identifying opportunities for intervention to shift these behaviors toward recommended levels [36,37].

Recent advancement of smart phone and wearable technology such as food diaries, activity applications, smart watches, accelerometers, and wearable cameras have allowed researchers to collect large amounts of time-stamped data on multiple dietary and 24-h movement behaviors and their accompanying contextual factors [33,38]. These data facilitate the examination of temporal patterns but require advancements in analytic methods. Although a range of methods exist for analyzing the clustering of behaviors [[25], [26], [27]], they have mostly been applied to aggregated data whereby the temporal information about behaviors is lost. Broadly, these methods aim to uncover underlying patterns in the data by reducing high-dimensional data (i.e., number of observation features/covariates is larger than the number of subjects) into a smaller set of variables in a way that retains the variance in the original data [28,[39], [40], [41]]. Identification of methods suitable for analyzing temporal behavior patterns is a critical first step to advance understanding of complex lifestyles in relation to health. Therefore, the aim of this scoping review was to explore analytic methods for understanding the temporal patterning of multiple dietary and 24-h movement behaviors. Additionally, we explored methods that have incorporated contextual factors in the examination of temporal behavior patterns.

## Methods

This scoping review was conducted according to the Preferred Reporting Items for Systematic Reviews and Meta-Analyses extension for Scoping Reviews (PRISMA-ScR) guidelines [42]. The protocol for this review can be accessed via the Open Science Framework at https://osf.io/7fg98/ [43].

### Search strategy

An initial literature search was conducted on 3 May, 2021, in the Embase, ProQuest, and EBSCOhost (PsycINFO, MEDLINE Complete, SportDiscus) electronic databases. The search strategy involved consultation with the institute librarian to ensure maximum coverage of key platforms such as PubMed and included peer-reviewed human studies published in English between 1 January, 1997, and 30 March, 2021. The year 1997 was chosen as the search start date because studies using real-time technology (e.g., accelerometers, electronic food diaries) to assess diet and 24-h movement behaviors became more prominent in the late 1990s and early 2000s [38,44]. Keywords used for the literature search covered 4 main concepts, including dietary and movement behaviors, behavior context, temporal patterning, and novel analytic methods (Supplemental Table 1). Once the key terms were identified and refined, searches were completed for each of the databases and, where necessary, modified to accommodate the individual databases. An updated literature search using the same databases, search, and screening strategies was conducted in July 2022.

### Eligibility criteria and article selection procedures

Supplemental Table 2 provides a summary of the study eligibility criteria. This review included peer-reviewed cross-sectional, prospective cohort, and intervention studies conducted in children and/or adults. To identify methods for determining temporal patterns based on combinations of behaviors, studies were required to have temporal information (i.e., time series data) on ≥2 of the 4 behaviors (diet, sleep, SED, PA). To identify methods for incorporating contextual factors into temporal behavior patterns, temporal information on ≥1 behavior and accompanying contextual information about that behavior (e.g., dietary intake and location of eating) was required. Articles were included if they used multivariate methods that analyzed the temporal behavior and context data as an integrated pattern. For example, studies were ineligible if they examined individual behaviors separately, across different models, or analyzed data in a way whereby the timing of the behavior was not incorporated. Articles were excluded if they did not involve humans, were not in English, were reviews, qualitative studies, or conference abstracts, focused on a single 24-h movement or dietary behavior only (i.e., did not include multiple behaviors or additional contextual factors).

On completion of the literature search, duplicates were removed and 2 reviewers (SEC and RML) screened all titles and abstracts of the remaining identified articles in Covidence [45]. Following this, full-text screening was also performed independently by SEC and RML. For approximately a third of the full-texts, uncertainties about their inclusion were resolved through discussion of the inclusion and exclusion criteria between reviewers (Supplemental Table 3). The reference lists of the included full-text articles and recent reviews on behavior patterns [7,22,46] were also searched for relevant articles.

### Data extraction and synthesis of results

Data extraction and entry of items into an Excel spreadsheet were conducted independently by 2 members of the review team (SEC and RML). First, the following information on study characteristics were extracted and included: author, year, study design and duration, study population (sample size, recruitment location, age, sex, health status), behaviors assessed, measurement of behaviors (assessment tools and duration, methods to classify levels of behaviors), contextual factors assessed, and measurement of contextual factors. Second, the following information on the methods used to assess temporal patterning were extracted: analysis aim, data structure (units, clustered/nested, time segmentation), analytic sample size, analysis input variables (number, time segmentation, units, type of variables), details of the analytic method, software used, measures of model robustness, findings on the number and type of temporal patterns and any associations with health outcomes where reported, data visualization methods used to present findings, and observations on the strengths and limitations of the analysis methods. Following data extraction, the study findings were summarized and narratively described.

## Results

### Search results

Supplemental Figure 1 shows a PRISMA flow diagram of the study selection process. The database searching and citation searching identified 32,607 (initial search), 6485 (updated search), and 3 (citation search) articles. After removing duplicates, 27,199 (initial search) and 6093 (updated search) article titles and abstracts were subsequently screened, and of these, 135 were assessed based on their full-text for eligibility. Following screening, 14 studies were included in this review.

### Study characteristics

Of the 14 included studies, 4 examined temporal patterns based on 2 or more behaviors, without contextual factors (Table 1 [[47], [48], [49], [50], [51]]). Study years ranged from 2017 to 2022 and were conducted in Europe [47,51], the United States of America [49], New Zealand, and 10 countries spanning Europe, North America, South America, and Australia [50]. All but 1 longitudinal analyses of a prospective cohort study [47] were cross-sectional. One study included children and adolescents from the Gateshead Millenium Study [47], whereas the 3 studies included adults only, of which 1 included older adult patients with chronic obstructive pulmonary disease [50]. All studies included males and females, with the percentage of male participants ranging from 42% to 65%. Three of the 4 studies examined multiple waking behaviors (e.g., PA, including different PA intensities, and SED), whereas only 1 study focused on both dietary and waking behaviors [49].

**TABLE 1:** Characteristics of individual studies examining temporal behavior patterns based on 2 or more behaviors only (i.e., no contextual factors).

Reference	Country; recruitment location; study name	Study design; data collection periods^1^	Study population	Behaviors assessed	Description of behaviors	Measures used to assess behaviors
Farooq et al. [47,48]	England; Northeast England; Gateshead Millennium Study	P 2006–2007, 2008–2009, 2011–2012, 2014–2015	*n* = 672 (analytic sample) Age = 7, 9, 12, and 15 y at each respective period Male = 49.6% Obese at age 15 = 14%	PA, SED	Daily MVPA (Evenson cut points); daily SED (<100 cpm)	Accelerometer (ActiGraph); 15-s epochs; worn minimum 2 weekdays and 1 weekend day for >6 h
Lin et al. [49]	USA; NHANES 2003–2006 (2 survey cycles)	C/S 2003–2006	See Supplemental Figure 1; analytic sample = 1836 Age groups 20–34 y = 29.4% 35–49 y = 38.2% 50–65 y = 32.4% Male = 49.5%	Diet, PA	Start time and energy intake per minute of each EO^2^ and total daily energy intake; vertical accelerations recorded as PA counts per minute (relative intensity per minute)	24-h dietary recall (valid weekday); ActiGraph accelerometer (random valid weekday of >10 h/d wear time)
Mesquita et al. [50]	Leicester, London, Liverpool, Horn, Eindhoven, Wiesbaden, Hamburg, Berlin, Lübeck, Hannover, Grosshansdorf, Frankfurt, Alicante, Madrid, Barcelona, Catalonia, Euskadi and Balearic regions, Dublin, Basel, Zürich, Pisa, Rochester, Londrina, Sydney, Perth	C/S Varies by data source but covers years 2004–2013	*n* = 1001 (analytic sample reported only) Age = 67 y; range = 61–72 y Male = 65% Patients with chronic obstructive pulmonary disease Mean BMI = 25.8	PA; SED	PA: LPA (2.0–2.9 METs) and MVPA (≥3.0 METs) SED: <2.0 METs	SenseWear Armband/Mini Armband, worn for ≥22 h/d over a minimum of 4 d, including Saturday and Sunday
Niemelä et al. [51]	Finland; Northern Finland; Northern Finland Birth Cohort 1966 Study	C/S 2012–2014	n = 6851 Age = 46 y Male = 42%	PA, SED	Daily LPA (2–3.49 METs) and MVPA (≥3.5 METS); Daily SED (1–1.99 METs)	Uniaxial accelerometer (Polar Active); 30-s epochs; worn 24 h for 14 d

Abbreviations: BMI, body mass index; C/S, cross-sectional; diet, dietary behavior; EO, eating occasion; GIS, geographic information system; LPA, light physical activity; MET, metabolic equivalent; MVPA, moderate-to-vigorous intensity physical activity; NR, not reported; P, prospective; PA, physical activity; SED, sedentary behavior.

1 For prospective studies, the baseline and follow-up years of data collection are shown.

2 Duration of eating was not recorded, so all EOs were standardized to be 15-min long.

PA and SED assessment tools included accelerometers: ActiGraph [47,49], Polar Active [51], and SenseWear armband/mini armbands [50]. Device wear time mostly ranged from 3 d, including the weekend [47], to 14 d [51]. Lin et al. [49] used a random valid weekday, defined as >10 h of wear time, from ActiGraph data collected over 7 consecutive days. The cut points used to classify PA intensities in adults varied but were mostly <2.0 metabolic equivalent of tasks (i.e., an estimate of the ratio of a person’s energy expenditure rate during a specific PA, relative to energy expenditure while resting) used by the body during PA for classifying SED [50,51]. Evenson cut points were used to classify both PA intensities and SED in the study of children [47]. In the study by Lin et al. [49] that examined dietary behaviors with PA, energy intake at eating occasions defined as an eating window of 15 min was assessed using a 24-h weekday dietary recall.

Of the 14 included studies, 10 of examined 1 or more 24-h movement [48,[52], [53], [54], [55], [56], [57], [58]] or dietary behaviors [59,60] together with contextual factors (Table 2). Study years ranged from 2009 to 2022, with all but 2 [59, 60] published within the last 8 y. Studies were mainly conducted in Europe (*n* = 6) [48,54,56,[58], [59], [60]] and the United States of America [52,55,57]. Five of the 10 studies analyzed data from prospective cohort studies [48,52,[55], [56], [57]]. Four studies included children and adolescents with ages ranging between 7 and 18 y [48,53,56,58], and 1 prospective study followed children aged 14 y for 9 y into their early adulthood [57]. The remaining 6 studies included adults, of which 2 included young adults (i.e., college/university students) [52,55]. All studies included males and females except for 1 female-only study [57].

**TABLE 2:** Characteristics of individual studies examining temporal behavior patterns together with contextual factors.

Reference	Country; recruitment location; study name	Study design; data collection periods^1^	Study population	Behaviors assessed	Description of behaviors	Measures used to assess behaviors	Contextual factors assessed	Measures used to assess contextual factors
Farooq et al. [48]	England; Northeast England; Gateshead Millennium Study	P 2006–2007, 2008–2009, 2011–2012, 2014–2015	*n* = 671 (analytic sample) Age = 7, 9, 12, and 15 y at each respective period Male = 49.6%	PA, SED	Daily MVPA (Evenson cut points); daily SED (<100 cpm)	Accelerometer (ActiGraph); 15-s epochs; worn minimum 2 weekdays and 1 weekend day for >6 h	Outdoor play, commuting to school, participation in after school sports clubs and availability of safe places for play at age 7. School sports clubs’ participation	Questionnaire
Farrahi et al. [54]	Finland; Northern Finland; Northern Finland Birth Cohort 1966 study	C/S 2012–2014	*n* = 5621 Age = 46 y Male = 42% Mean (SD) BMI = 26.7 (4.8)	PA, SED	Daily LPA (2–3.49 METs) and MVPA (≥3.5 METs); daily SED (1–1.99 METs)	Uniaxial accelerometer (Polar Active); 30-s epochs; worn 24 h for 14 d	Built environment (13 variables); natural environment (4 variables); socioeconomic environment (22 variables)	Residential coordinates. ArcGIS 10.3 was used to calculate built, natural, and socioeconomic environment variables (1-km buffer)
Gába et al. [58]	Czech Republic; urban and rural primary and secondary schools across the Czech Republic	C/S NR	*n* = 907 Age = 8–18 y Male = 42%	PA, SED, sleep	Vector magnitude activity counts per 5 s were used to estimate SED (<306), LPA (306–817), MPA (818–1968), and VPA (≥1969). Sleep duration was derived using the Cole–Kripke algorithm	Accelerometer (ActiGraph wGT3X-BT or GT3X); raw data reintegrated to 5-s epochs; worn 7 consecutive days	School hours and out-of-school hours	Participant log diaries
Jaeger et al. [59]	Spain; all regions and islands of Spain (sample biased toward major cities)	C/S NR	*n* = 831 Age = 25–49 y 53.7 male NR	Diet	Time and type of EO consumed the previous day (selected from 13 EO options) and the main food and/or drink component of each EO	A purpose-designed online 24-h recall survey instrument; 1 recall completed	No. of previous meals eaten that day; whether meal was cold, warm, mix of warm/cold items; location (including specific details, e.g., room of house), activity while eating, who prepared it	A purpose-designed online 24-h recall survey instrument
Parker et al. [56]	Spain; Primary and secondary schools from Cadiz and Madrid regions; UP and DOWN study	P 2011–2012, 2012–2013, 2013–2014	*n* = 1188 children, *n* = 1037 adolescents Male =51% Mean (SD) age = 11.9 (2.5) y at baseline OW/OB = 33%	PA, SED	MVPA (≤2000 cpm); SED (<100 cpm) analyzed at 10-s epochs to derived average MVPA and SED min/d	GT1M, GT3X, and GT3X + ActiGraph accelerometers worn for 7 consecutive days (10 h/d for 3 d minimum wear time)	Items corresponding to subscales/scores on positive and negative effect, perceived pros and cons of reducing SB, independent mobility, screen time rules, co-participation in TV viewing and PA, parental modeling of TV viewing and PA, neighborhood and school environment conduciveness to PA, availability of PA and SED items	Questionnaire at baseline (reliability tested—reference provided)
Riou et al. [60]	France; Paris metropolitan area; 2010 Wave of the SIRS Cohort	C/S 2010	*n* = 3006	Diet	Intake of meals defined as eating events considered by participants themselves	Face-to-face survey, including detailed questions about meal structures in a typical week	Time period, meal location (home, working place, restaurant), with whom meal is taken (alone, family, colleagues/friends), activities with meals (TV, radio, computer, reading, chatting)	Face-to-face survey, including detailed questions about meal structures
Vidal Bustamante et al. [55]	USA; Harvard University	P NR but 1 academic year duration	*n* = 68 Age range: 18–19 y Male = 49%; Previous diagnosis of a psychiatric disorder	Sleep	Sleep duration (h), sleep timing regularity (% of overlap in sleep timing between each study day and average sleep timing), and wake-time activity (time between sleep episodes in consecutive days estimated using DPSleep) and major sleep episodes (DPSleep^2^)	Triaxial accelerometer (wrist) with timestamps at a rate of 30 Hz (10 Hz when not on campus). Participants indicated sleep start/end times by pressing a button on the wrist band	Items on previous day subjective sleep quality, energy levels, PA levels, awake time spent on schoolwork and socializing, feelings of stress, positive affect, and negative affect, and participants’ sources of stress. Psychological problems and clinical symptoms, perceived stress, anxiety, and depression in the past 2 wk	Daily phone-based surveys via the Beiwe application administered each night before bed and biweekly surveys on psychologic issues assessed using symptoms Checklist 90 Revised, PSS-14, GAD-7, PHQ-9^2^
Wang et al. [52]	USA; University of Notre Dame; NetHealth Study	P August 2015–May 2017	*n* = 730 Age = NR; college freshmen (i.e., first year at university) Male = 49.19% mean BMI = 22.82	PA, SED, sleep	daily PA: calories and minutes (low range, fat burn, cardio, peak), steps, floors, minutes (lightly active, fairly active, very active), marginal calories, activity calories, calories BMR, and calories out, daily steps, daily active minutes, daily activity calories; daily SED minutes; daily SLEEP minutes	Fitbit worn for ≥80% of a day—637 d. Sleeping minutes were estimated using an algorithm based on the movement pattern and heart rate variability	Peer influence, daily network size, daily in bed minutes, classes taken that day, on campus (yes/no), weather indicators (5 variables)	Smartphone communication records measured peer influence (daily in-study contacts) and network size (total number of contacts) that participant communicated with each day. Daily weather data indicators (6 variables) from website: http://www.usclimatedata.com/
Yang et al. [57]	USA; Maryland Field Center, Baltimore	P 2006–2006, 2009–2009, 2015–2015	*n* = 730 eighth-grade girls Age = 14, 17, and 23 y at each respective period Female = 100%	PA	MVPA (acceleration counts per 30 s that were >1500)	Actigraph accelerometer (Xsens MTi Series, model 7164) set to 30-s epochs worn for 7-consecutive days (minimum wear time = 4 d, 10 h/d)	Items/scales assessing self-management strategies, PA self-efficacy, PA enjoyment, perceived barriers to PA, PA outcome-expectancy, depressive symptoms, social support for PA, perceived neighborhood environmental factors	Questionnaire at each time point
Zhao et al. [53]	New Zealand; primary and intermediate schools in Auckland; Neighborhoods for Active Kids project	C/S NR but data collected over 1 wk	*n* = 1102 Age = boys: 10.66 y, girls: 10.53 y Male = 49.5%. OW/OB = 27.4%	PA, SED, sleep	LPA, MVPA, and SED: categorized using Evenson cut points Sleep: sleep and waking times	PA and SED: Actigraph GT3X+ worn on waist over 7 d Sleep diary (completed by parents)	Home location, regular travel mode to school (active or passive)	Internet-based survey tool, softGIS

Abbreviations: BMI, body mass index; BMR, basal metabolic rate; C/S, cross-sectional; diet, dietary behavior; EO, eating occasion; GIS, geographic information system; LPA, light physical activity; MET, metabolic equivalent; MVPA, moderate-to-vigorous intensity physical activity; NA, not assessed; NR, not reported; OW/OB = overweight or obesity; P, prospective; PA: physical activity; SED, sedentary behavior; SIRS: a French acronym for “Health, inequalities and social ruptures”; TV, television.

1 For prospective studies, the baseline and follow-up years of data collection are shown

2 Patient Health Questionnaire (PHQ) 9-item scale, Perceived Stress 14-item scale (PSS) , Generalized Anxiety Disorder (GAD) 7-item scale (minimum of 100 daily surveys across the year, completed within acceptable time window).

PA and SED assessment tools included accelerometers: ActiGraph [48,53,[55], [56], [57], [58]], Polar Active [54], and Fitbits [52]. Sleep was measured using ActiGraph accelerometers [55,58], Fitbits [52], daily phone-based surveys (i.e., using principals of ecological momentary assessment) [55] and diaries (parent-completed) [53]. Device wear time mostly ranged from 3 d [48,56] to 14 d [54]; however, the study by Vidal Bustamante et al. [55] and Wang et al. [52] included 256 d of ActiGraph and 637 d of Fitbit data, respectively.

The contextual factors examined in the studies of 24-h movement behaviors varied and broadly included factors relating to the built, natural, and socioeconomic environment [48,54,56,57]; meteorologic (temperature, precipitation) data [52]; daylight hours [52]; school hours [58]; perceived stress [52,55]; sleep quality [55] and energy levels [55]; sources of stress [55]; positive and negative effects [55,56]; symptoms of depression [55,57]; anxiety and stress [55]; self-management strategies [57]; perceived barriers to PA [57]; social support for PA [57]; perceived self-efficacy and enjoyment of PA [57]; home location [53]; activity interests [48], parental modeling or co-participation [48,56]; screen time rules [56]; and mode of transport to school [48,53]. Contextual factors were assessed using residential coordinates and geographic information system (i.e., map software that analyzes geographic location) [53,54], smartphone communication and geolocation records [52], daily weather data (website) [52], college course data [52], Fitbit data [53], log diaries [58], and survey tools [48,52,53,[55], [56], [57]]. In the 2 studies on dietary behaviors and contextual factors, both examined eating occasions, which included meals and snacks [59] and meals only [60]. Contextual factors included meal location [59,60], presence of others [60] and activities while eating [59,60], meal temperature (warm, cold, or mixed), who prepared the meal, and number of previous meals eaten that day [59]. Diet and contextual factors were assessed using a purpose-built online 24-h recall instrument [59] and a face-to face diet history survey that included detailed questions about meal structures and characteristics in a typical week [60].

### Analytic methods used across the included studies

TABLE 3, TABLE 4 presents summarized findings from individual studies examining temporal behavior pattern (without and with contextual factors, respectively) and their analytic approaches. Supplemental Table 3 provides a more detailed description of the analytic approaches used in each study. Of the 4 studies examining multiple behaviors only (i.e., PA and SED; diet and PA), 1 study used group-based multitrajectory models to examine long-term temporal patterns (i.e., yearly patterns) [47] whereas 3 studies used 1 or more machine learning approaches to examine daily temporal patterns [[49], [50], [51]]. For example, a combination of distance-based clustering algorithms was applied to multidimensional accelerometer and dietary time series data by Lin et al. [49] and principal component analysis (PCA) and k-means cluster analysis were applied to 180 continuous waking behavior variables by Mesquita et al. [50].

**TABLE 3:** Findings of individual studies examining temporal behavior patterns based on 2 or more behaviors only (i.e., no contextual factors).

Reference	Aim of analysis	Analytic sample (*n*)	Data structure	Analysis input variables	Analytic approach	Data visualization methods	Selected temporal pattern findings	Selected associations with health-related outcomes
Farooq et al. [47]	To identify multitrajectory latent groups for time spent in MVPA and SED jointly across childhood and adolescence	602	Time series: average time spent in MVPA (min/d) and SED (min/d) across 4 time points over 8 y	Continuous variables of MVPA and SED (min/d) at each time point	Group-based multitrajectory analysis	Line graphs of mean time (95% CI) spent in MVPA and SED simultaneously at each time point, by latent trajectory group	3 patterns: inactive during childhood and adolescence (31%), active during childhood only (54.2%), and active during childhood and adolescence (14.8%)	FMI was higher at age 15 y in the inactive group, than that in the most active group
Lin et al. [49]	To create clusters of joint temporal dietary and PA patterns and examine their associations with health outcomes	1836	2D diet and PA time series: minutes over length 1440 (24 h × 60 min)	2D vectors consisting of EI and PAC at the *i*th minute of the day	Distance-based clustering analysis (dynamic time warping)	Heat maps of the distribution of nonzero EO and PAC over 24 h, by cluster. Color highlighted the highest EI and PA counts	4 patterns: *1*) highest PA, 3 evenly spaced EO with higher EI in noon and evening; *2*) lowest PA and more energy equivalent EO with no distinct EI peaks; *3*) evening PA pattern; *4*) early morning PA pattern that decreased across the day, highest absolute EI concentrated in noon and evening	Adults with pattern 1 had lower BMI, WC, and total cholesterol concentration than those in pattern 4
Mesquita et al. [50]	To describe PA measures and PA hourly patterns in patients with COPD after stratification for generic and COPD-specific characteristics and to identify clusters of patients with COPD based on PA measures	1001	Minute-by-minute energy expenditure and METs data during waking hours, covering a minimum of 2 weekdays, Saturday, and Sunday	180 PA variables (10-min bouts in same intensity); time (before/after 12:00); frequency (bouts per day); and quantity (absolute numbers or percentage of total)	Principal component analysis (PCA) and k-means cluster analysis	Graph in 2 or 3 dimensions presenting the 2 or 3 PCA components by cluster membership; daily PA intensity (METS) hourly pattern by cluster membership	5 patterns: *1*) couch potatoes (22%); *2*) highly sedentary (41%); *3*) sedentary movers (18%); *4*) sedentary exercisers (17%); and *5*) busy bees (2%). For all patterns, peak of PA intensity occurred before midday	Couch potatoes were characterized by higher BMI, lower FEV1, worse dyspnea, and higher ADO index (age, dyspnea, and airflow obstruction) than other clusters
Niemelä et al. [51]	To identify the intensity and temporal patterns of PA and SED and their relationship with CVD risk	4582	Time series: 10-min averages of 30-s MET data over 7 consecutive valid days^1^, including weekend days	1008 MET summary variables (144 per day)	Clustering (decision trees using X-means algorithm)	Radar chart of standardized cluster means (*z*-scores) of PA intensity levels in clusters. Line graphs of average MET by time of day	4 patterns: *1*) inactive (41.1%), *2*) evening active (17.5%); *3*) moderately active, (28.3%); and *4*) very active—mostly during day (13.1%)	The inactive cluster had higher risk of developing CVD in next 10 y compared with the very active cluster and moderately active (women only) clusters

Abbreviations: ADO, age dyspnoea (airflow) obstruction; BMI, body mass index; COPD, chronic obstructive pulmonary disease; CVD, cardiovascular disease; EI, energy intake; FEV1, forced expiratory volume in the first second; FMI, fat mass index; LPA, light physical activity; MET, metabolic equivalent; MVPA, moderate-to-vigorous intensity physical activity; NA, not assessed; NR, not reported; OW/OB, overweight or obesity; PA, physical activity; PAC, physical activity counts; SED, sedentary behavior; WC, waist circumference.

1 600-min wear time constituted a valid day.

**TABLE 4:** Findings of individual studies examining temporal behaviors patterns together with contextual factors.

Reference	Aim of analysis	Analytic sample (*n*)	Data structure	Analysis input variables	Analytic approach	Data visualization methods	Selected temporal pattern findings	Selected associations with health-related outcomes
Farooq et al. [48]	To examine factors associated with trajectories of MVPA and SB across childhood and adolescence	671	Time series: average time spent in MVPA (min/d) and SED (min/d) across 4 time points over 8 y	Continuous variables of MVPA and SED (min/d) at each time point	Group-based multitrajectory analysis	Line graphs of mean time (95% CI) spent in MVPA and SED simultaneously at each time point, by latent trajectory group	Same patterns as per Farooq et al. [47]. Commuting to school, safe play spaces, and participation in sports co-patterned with the most active group over time	NA
Farrahi et al. [54]	To classify individuals’ PA behavior using individual, demographical, psychological, behavioral, environmental, and physical predictors	4582	Time series: 10-min averages of 30-s MET data over 7 d, including weekend days	1008 MET summary variables and 168 predictor variables	Clustering (decision trees using X-means and CHAID algorithms)	The final decision tree showing the percentage of active and inactive participants in each subgroup	After CHAID, 54 subgroups of active/inactive participants with differing PA accumulation patterns were identified. Most active subgroups had lower SED level than population mean, some had higher MVPA and others higher LPA	Participants with higher body fat (>31%) were more likely to be inactive, than those with lower body fat percentage. (<28.3%)
Gába et al. [58]	To estimate compositional associations between context-specific SED adiposity and the effect of replacing school and out-of-school SED with PA	336	Time series: vector magnitude activity counts per 5 s and nightly hours of sleep duration over 7 consecutive valid days^1^	Sum of the average time spent in SB, LPA, MPA, and VPA during school and out-of-school hours, and sleep normalized to 24 h (i.e., 1440 min)	Compositional data analysis	Line graphs of estimated relative difference in fat mass percentage with replacing school and out-of-school SED with LPA	Girls and boys spent 64%–69% of school and out-of-school hours in SED, but boys spent more school time in VPA and less time in out-of-school LPA and MPA than girls	Replacing 30 min/d of out-of-school SB with PA was associated with 14% lower FMI in girls only
Jaeger et al. [59]	To examine the extent to which eating is organized over time and space and influenced by who is present and to provide descriptions of ways in which eating is organized	8231	Time-series data: time, food content, and contextual characteristics of eating occasions over 24 h	21 food groups (binary), contextual characteristics (number NR) age, sex, education	Canonical correspondence analysis	Canonical correspondence map	2 patterns: *1*) eating occasions structured by the time of day (e.g., meal 1 took place early in the day and involved cereal, morning bakery pastry); *2*) eating occasions structured by formality indicators (e.g., more formal meals involved sitting, hot carbs/meat, taken in the afternoon at home, sitting with friends or family)	NA
Parker et al. [56]	To identify group-based dual trajectories of PA and SED and their correlates	1597	Time series: accelerometer data, collected over 7 d, were analyzed in 10-s epochs and used to compute mean MVPA and SED min/d. Minimum wear time was 10 h/d for 3 d	Average MVPA and SED (min/d) from 3 time points: 2011–2012, 2012–2013, and 2013–2014	Parallel process growth mixture models with regressions among the random-effects, followed by multinomial linear regression	Lines graphs of the estimated linear trajectories for MVPA and SED by group membership	4 patterns: *1*) stable MVPA and decreasing SED with parents (4%); *2*) stable MVPA and increasing SED (3%); *3*) consistently higher MVPA over time (18%); and *4*) stable low MPVA with slightly increasing SED (75%). Parental screen time rules and father modeling TV viewing were linked with patterns 1 and 4, respectively	Pattern 1 was associated with lower scores on the negative affect scale than pattern 3
Riou et al. [60]	To examine the so-called French model of eating behavior in adults in the Paris area, by identifying and characterizing distinct meal patterns	2994	Time series: time, food content and contextual characteristics of meals in a typical week	Mealtime period (6 variables); meal frequency; contextual characteristics (3 variables)	Clustering (partitioning around medoïds algorithm with Manhattan distance)	None	5 patterns: *1*) 3 meals, often outside (33%); *2*) 3 meals, mostly alone at home (17%); *3*) 3 meals, at home with family (24%); *4*) 1 or 2 meals, mostly at home with television (13%); and *5*) 2 meals, often outside (12%)	Patterns 4 and 5 were associated with higher daily snacking, dissatisfaction concerning food, and food quality affected by lifestyle
Vidal Bustamante et al. [55]	To examine and characterize first-year college students’ affective and behavioral experiences throughout the academic year	49	Time series (256 d): phone-based survey data on daily affective, sleep, social, academic, and stress experiences in were summarized into daily-level composite, binary, or summary scores. Raw actigraphy data were used to estimate minute-based PA, sleep duration, and sleep timing regularity using DPSleep processing pipeline	Group-averaged time series data averaged at the daily level over 256 d. Variables were sleep duration, sleep regularity, sleep quality, PA, energy, time spent on schoolwork, time spent on social interaction, procrastination, feeling connected to others, negative affect, stress, number of stressors, academic stress, social stress, and status stress	Latent profile analysis, followed by Hidden Markov modeling to examine latent distress states over time across clusters and mixed-effects models or ANOVAs to examine predictors of clinical symptoms	Biplots showing the maximal separation among the profiles in relation to the original input variables. Bar or box plots showing between person-means for variables, stratified by profile. Sequence/time series graphs of latent states of distress and affective/stress states over year for each student profile	3 patterns: *1*) lowest negative affect, stress levels, and time spent on schoolwork, highest levels of PA (24%); *2*) moderate stress levels (except academic, which was high), highest sleep duration, and lowest PA (35%); and *3*) most negative affect, highest academic and social stress levels, lowest sleep quality and energy, and highest procrastination (41%)	Pattern 1 was in low or medium distress states almost all year; pattern 2 was associated with a medium distress state throughout most of the academic year and had the highest academic grades; pattern 3 was associated with higher Global Clinical Scores and had highest distress states during school semesters
Wang et al. [52]	To examine how temporal growth trends in PA respond to changing social, environmental, and behavioral characteristics	619	Minute-by-minute movement and heart rate data aggregated into 637 d of daily activity estimates	Daily PA status was a standardized factor score of 18 PA Fitbit items. Predictors of PA included 14 time-varying contextual factors	Factor analysis, followed by latent growth-curve models with random/fixed effects	NR	4 patterns: *1*) peer influenced: PA increases in tandem with PA levels of daily contacts; *2*) social activity predicted more PA; *3*) behavioral: more sleep predicted higher daily PA; *4*) environmental: being on campus and more daily classes, Saturday and days with highest temperature were most active	NA
Yang et al. [57]	To examine factors related to PA trajectories among adolescent girls	568	Time series: 7 days (minimum wear time <days, 10 h/d) of accelerometer data were used to calculate daily minutes in MVPA at each time point. Individual-, social-, and neighborhood-level variables from survey data at each time point	Daily MVPA at each time point (3 variables); 31 variables (time variable; individual-, social-, and neighborhood-level variables) were considered and initially entered into the model (16 were selected)	Model-based clustering using a mixture of linear mixed-effects models with double penalization on fixed effects and random effects	Line graphs of mean and individual MVPA trajectories by cluster	3 patterns—*1*) MVPA maintainers (42%): higher MVPA in this cluster was predicted by friend support and more parks in their neighborhood; *2*) decreasers (51%): higher levels of MVPA in this group was predicted by higher self-management strategies and self-efficacy and lower outcome-expectancy; *3*) increasers (7%): higher MVPA in this group predicted by self-management strategies, less neighborhood traffic, and longer distance to nearest park/school	BMI was not associated with any of the clusters
Zhao et al. [53]	To visualize compositional time-use patterns of children’s daily activities using visualization methods (ring maps, time-activity diagrams) which integrate space, time activity, neighborhood deprivation, and weight status to provide insights what would otherwise be inaccessible	882	Not clear. Accelerometers worn over 7 d were initialized to log raw data at 30 Hz. Time-stamped accelerometer data were then collapsed into 30-s epochs	Ring maps: the average percentage of weekday time spent on each activity (MVPA, LPA, SB, sleep) across eight 3-h time blocks, by neighborhood deprivation. Time-activity diagrams: mean percentage of weekday time (10-min intervals) in each of the 4 activities are summed to 100% for each individual and activities are overlaid where they occurred simultaneously	Ring maps and time-activity diagrams (data-driven visualization methods)	Ring maps and time-activity diagrams	Ring maps: Children from most deprived areas slept the least, started sleep later in the evening, were more sedentary during morning school hours, and spent more time in SED and LPA than peers from least deprived areas. Time-activity diagrams: In boys from the most deprived areas, MVPA and LPA occur during breaks in the weekday and just before and after school, sleep started as early as 19:00 for some boys and SED dominates the time between 17:00 and 20:00, especially among those OW/OB	Ring maps: In the time block from 21:00 to 00:00, boys with OW/OB in the most deprived areas slept less and spent more time in LPA and SB than their counterparts in the least deprived areas. Time-activity diagrams: Boys from deprived areas with OW/OB spent more time in SED throughout the day, especially in the period immediately after school

Abbreviations: ANOVA, analysis of variance; BMI, body mass index; CHAID, chi2 automatic interaction detection; FMI, fat mass index; LPA, light physical activity; MET, metabolic equivalent; MVPA, moderate-to-vigorous intensity physical activity; NA, not assessed; NR, not reported; OW/OB, overweight or obesity; PA, physical activity; SED, sedentary behavior; TV, television.

1 A valid day constituted ≥1200 min of a total wear time with ≥720 min of awake time, ≥320 min of sleep time, and 220–440 min of school time.

Ten studies examined dietary and/or 24-h movement behaviors together with contextual factors (Table 4 [48,[52], [53], [54], [55], [56], [57], [58], [59], [60]]). Vidal Bustamente et al. [55] and Yang et al. [57] used model-based clustering techniques (e.g., latent profile analysis) to determine sleep and PA patterns with contextual factors, respectively. In the study by Yang et al. [57], model-based clustering was used in conjunction with linear mixed-effect models to simultaneously determine cluster patterns and their predictor variables as part of a novel 1-step clustering procedure [57]. For studies examining multiple 24-h movement behaviors with contextual factors (i.e., PA and SED; PA, SED, and sleep), 2 used a type of model-based clustering for longitudinal data (i.e., data with 3 time points or more) to determine joint temporal patterns for multiple behaviors over longer durations [48,56].

Most of the included studies on PA and SED used a combination of approaches to determine daily temporal patterns and their contextual correlates [[52], [53], [54]]. For example, Farrahi et al. [54] used 2 clustering approaches to identify the temporal patterns of PA intensities and SED in relation to cardiovascular disease [51] before classifying the patterns according to 168 different contextual factors[54]. Wang et al. [52] used latent growth-curve models to examine temporal growth trends in PA patterns first derived from PCA in relation to changing contextual factors over 637 d.

Two studies on children used compositional data analysis and data-driven visualization methods, specifically ring maps and time-activity diagrams [53], to understand compositional 24-h movement data and facilitate comparisons between groups and contexts. Of the 2 studies examining dietary behaviors only, 1 applied canonical correspondence analysis to food group and contextual variables to examine how eating is structured over time and space [59]. The other study used a clustering algorithm to identify and characterize Parisian meal patterns from detailed interview data on mealtimes, meal frequency, and meal contextual factors [60].

### Temporal behavior patterns identified across the studies

The number of temporal patterns identified across the 40 studies on multiple behaviors without contextual factors ranged between 3 [47] and 5 [50] (Table 3 [47,[49], [50], [51]]). Most of these studies reported statistical measures used to determine the number of patterns [47,49,50] (Supplemental Table 3). Using PA and SED behaviors, Niemelä et al. [51] found 4 patterns labeled inactive, evening active, moderately active, and very active—mostly during day, with the inactive cluster having the highest risk of cardiovascular disease in the next 10 y, than the very active cluster [51]. Mesquita et al. [50] identified 5 patterns in older patients with chronic obstructive pulmonary disease, 4 of which were characterized by varying degrees of SED [50]. Of note, across all patterns, PA intensity was the highest before midday, however, “couch potatoes” had the poorest health risk profile than other patterns. Farooq et al. [47] found 3 patterns of joint PA and SED trajectories characterized by being inactive [i.e., <60 min moderate-to-vigorous intensity physical activity (MVPA) and 400–600 min SED per day] during childhood and adolescence, active during childhood only, and active during childhood and adolescence. Fat mass index was the highest in the inactive group at age 15 [47].

Four temporal patterns were identified in the 1 study examining dietary and waking behaviors [49]. The first 2 patterns comprised more women and were characterized by *1*) low PA and low variation in energy content across the main eating occasions (i.e., no distinct peaks of energy intake) and *2*) an evening PA pattern also with low variation in energy content across main eating occasions. The remaining 2 patterns comprised more men, the first of which included an early morning PA pattern with the highest overall energy intake disperse across the noon and evening periods. The second was a high overall PA pattern (only 14% of participants) with 3 evenly spaced eating occasions that were also higher in energy intake in the afternoon and evening. Adults with the high overall PA pattern or the evening PA pattern had lower BMI (in kg/m^2^) and waist circumference than those with the low PA pattern or the early morning PA pattern, with the most favorable estimates observed for the high overall PA pattern. Patterns were visually represented using heat maps of the distribution of energy-containing eating occasions and PA counts greater than zero [49].

The number of temporal patterns identified across the 10 studies on dietary and/or 24-h movement behaviors with contextual factors ranged between 3 [48,55,57] and 54 [54]. Seven of these studies reported the statistical indicators or metrics used to determine the number of patterns [48,52,[55], [56], [57]], however, the actual values for the statistical indicators by pattern number were rarely reported [52,56].

Among the studies on 24-h movement behaviors, Vidal Bustamente et al. [55] identified 3 sleep patterns linked to daily contextual factors and were characterized by the following: *1*) high sleep quality, low stress, low negative affect, and higher subjective energy and PA levels; *2*) highest sleep duration, regularity and quality, moderate stress, and lowest subjective PA levels; and *3*) poor sleep quality, high stress, and high negative affect. The latter pattern was associated with higher Global Clinical Severity Scores for psychologic distress [55]. Yang et al. [57] identified 3 groups of young adults who had consistently low MVPA between the ages of 14 and 23 y (maintainers), high MVPA at age 14 y but decreased over time (decreasers), or low MVPA at age 14 y but increased over time (increasers), and in each group, higher MVPA was predicted by unique contextual factors [57]. For example, in the maintainers group, MVPA was predicted by friend support and more neighborhood parks, but self-efficacy and self-management strategies were important predictors of higher MVPA in the decreasers group.

In the study by Farooq et al. [48], having a safe playing environment and actively commuting to school during childhood and sports club participation in adolescence predicted the “active” trajectory pattern (>60 min MVPA and <400–600 min SED per day) during childhood and adolescence [48]. In a similar study of joint SED and PA trajectories in children and adolescents, Parker et al. [56] found 4 distinct patterns but most participants (75%) had consistently low MVPA (<50 min/d) and increasing SED (∼740 min/d in year 1; 800 min/d in year 3), which was predicted by father modeling of TV viewing. In the same study, only 4% had stable MVPA over 3 y (∼49 to 57 min/d) and decreasing SED (1000 to 820 min/d), which was linked to parental screen time rules, and children with highest MVPA over time (>70 min/d) had lower scores for negative affect [56].

Farrahi et al. [54] identified 54 subgroups of participants with active and inactive temporal patterns—originally identified in the study by Niemelä et al. [51]—which differed by their waking behavior accumulation patterns [54]. Contextual factors that helped predict these subgroup patterns included weekday computer use time, sitting time at the workplace, number of public transport stops, and housing density [54].

Wang et al. [52] identified 4 key contextual factors that positively influenced college freshmen’s daily PA patterns and included sleep, peer influence, social activity, being on campus, and temperature [52]. Sleep also influenced daily 24-h movement behavior patterns in the study by Zhao et al. [53]; children from the most deprived neighborhood areas slept the least, went to sleep later, and were more sedentary in the morning school hours, than their counterparts in the least deprived areas. This pattern of less sleep and higher SED was also more prevalent in overweight or obese boys from deprived areas. Gába et al. [58] also reported sex-specific 24-h movement patterns. Although both boys and girls spent approximately two thirds of their school and out-of-school time in SED, boys spent more school time in vigorous-intensity PA and less time in out-of-school light-intensity PA, than girls [58]. In girls only, replacing 30 min/d of out-of-school SED was associated with 14% lower fat mass index in compositional isotemporal substitution models [58].

Two [59] and 5 [60] temporal patterns were observed in the 2 studies of dietary behaviors with contextual factors. Both studies used objective statistical indicators to guide the selection of patters. Jaeger et al. [59] found 2 eating patterns using canonical correspondence analysis, which were structured by time of the day (e.g., bakery pastries and cereals more evident in the morning) and indicators of formality (e.g., sitting, social setting). For example, meals involving sitting with others usually included hot carbohydrates and proteins and took place in the houses of family/friends. In contrast, “casual eating” tended to involve sweets, chocolates, or sandwiches and take place at the office, while working on the computer or other locations. Five distinct meal patterns were identified by Riou et al. [60], of which 3 were characterized by 3 meals per day within the conventional time periods for French adults but differed by contextual factors such as where (home, outside) and with whom (family, alone) the meal was eaten. The 2 remaining patterns were characterized by the omission of breakfast (i.e., a 2 meal per day pattern) and meals were consumed mostly at home with the TV or outside often, respectively.

### Visualization of temporal pattern data

A range of methods were used to visualize patterns across the included studies (TABLE 3, TABLE 4). Line graphs were the most common data visualization method used to present temporal patterns [47,48,50,[56], [57], [58]], and other methods included final decision trees [33], radar charts [34], biplots showing differences between patterns [55], sequence/time series graphs [55], 2D and 3-dimensional graphs of extracted PCA components [50], and ring maps and time-activity diagrams [53], and canonical correspondence maps [59].

## Discussion

We conducted a comprehensive scoping review to explore analytic methods used by researchers to examine dietary and 24-h movement behaviors as an integrated temporal pattern, and their contextual correlates, where presented. Fourteen studies were included in this review, and of these, 11 were published in the last 5 y, highlighting that this is a rapidly developing area of research. Only 1 study [49] combined temporal data on both waking and dietary behaviors, despite their roles in health and well-being and the evidence on the patterning of these behaviors in children and adults [23,25,61,62]. Clustering methods were mostly used to determine temporal patterns. Although clustering methods aim to allocate observations into distinct groups, the data-driven nature of these methods and the types of specific patterns that are examined mean that patterns will vary by the study population and research aim (i.e., patterns are not generalizable). Studies also varied in the type of clustering used; some studies used probabilistic clustering methods (e.g., latent profile analysis, group-based trajectory analysis), while others used machine learning techniques with various clustering algorithms used across those studies. Further, some studies used a combination of data reduction methods. Together, these findings highlight the complexity involved with combining and analyzing time-series data for multiple behaviors and their contextual factors. Progressing analytic techniques for examining the temporal patterning of dietary [7] and 24-h movement behaviors [63], and the contexts in which they occur, may help us better understand health-promoting lifestyles and, subsequently, inform healthy eating and 24-h movement interventions.

The heterogeneity in the methods used to determine patterns and the behavioral and contextual input variables make the findings difficult to compare across studies. However, despite the diversity in types and range in number of patterns found, there were still several observations of note when comparing the included studies that examined waking behaviors. For example, temporal patterns characterized by high SED and low PA were associated with indicators of higher adiposity in children and adolescents [47,53,58], and higher adiposity [50,54] and risk of cardiovascular disease [51] in adults. Although the studies differed in the periods examined—for example, 2–3 yearly [47] compared with daily/weekly [51,53,54,58] temporal patterns, the findings are in line with previous literature that show patterns characterized by high SED are associated with higher adiposity in youth [23,25,26]. Intervention studies in at-risk adults have also shown favorable changes in metabolic risk factors when breaking up prolonged SED time at a desk with regular brief bouts of PA [64,65]. However, the examination of temporal and contextual aspects of patterns allows us to characterize useful information about when, where, and how behaviors occur. For instance, both Gába et al. [58] and Zhao et al. [53] identified after school and evening hours as critical windows for reducing SED and increasing PA in boys [53] and girls [58]. In the study by Zhao et al. [53], weekday mornings were also identified as a critical window for PA intervention in boys with overweight/obesity living in the most deprived areas. For long-term and favorable PA and/or SED trajectories in children and adolescents, friend support or co-participation [56,57] and access to safe play areas or local parks [48,57] appear to be important factors and are supported by previous literature on PA/SED determinants [66,67]. These studies highlight that further investigation into temporal 24-h movement patterns on weekdays and weekend days may facilitate a more nuanced and meaningful understanding of how subpopulations use their daily time across different contexts and provide evidence for health-promoting interventions. Moreover, improved understanding of contextual determinants might provide insights into potential barriers and facilitators of behavior change, which are important considerations for the feasibility and effectiveness of behavior change interventions.

Very few studies included in this review examined the temporal patterning of dietary behaviors together with contextual factors (*n* = 2) or 24-h movement behaviors (*n* = 1). Although accelerometers collect time-stamped information on multiple 24-h movement behaviors across the intensity spectrum, they do not collect contextual data. Dietary assessment methods commonly used in nutrition research include food frequency questionnaires, which do not include information about the timing of eating occasions or their contextual factors. However, more recently, 24-h dietary recalls and food diaries, including some used in some national nutrition surveys, have been modified to collect both time-based or eating occasion-based dietary and contextual data, which enable the examination of temporal patterns [38]. The studies by Jaeger et al. [59] and Riou et al. [60] highlighted the role of context in food choices at eating occasions. For example, sandwiches, sweets, and chocolate were more commonly eaten when working on a computer, and fruit was more often eaten at the main meals later in the day in a sample of Spanish adults [59]. Furthermore, eating only 1 or 2 meals per day (with breakfast being the most skipped meal) at home with a TV or outside of home was linked to more snacking and lower dietary quality in French adults [60]. Neither of these studies examined associations with health outcomes; therefore, this is an area of investigation for future studies on temporal patterning of dietary behaviors.

Only 1 study was identified in this review that examined the temporal patterning of dietary and waking movement behaviors [49]. Lin et al. [49] found that adults with a pattern of high PA counts across the day (mean PA was 5.7 × 10^5^ counts/d) and evenly distributed energy intake at main eating occasions during the noon and evening was associated with more favorable cardiometabolic risk parameters, than adults with higher PA intensity in the morning and the high overall energy intakes dispersed at main eating occasions across the noon and evening. This latter pattern may indicate undesirable energy compensatory behaviors in response to the morning PA (i.e., unhealthy food or overeating as a reward for exercise) and highlights the potential impact of combining data on both dietary and 24-h movement behaviors. However, studies using multiple days of data, including weekend days, are required to elucidate the health effects of daily dietary and PA patterns [68]. Further, the lack of studies on the temporal patterning of dietary and 24-h movement behaviors may result from the practical challenges in collecting detailed longitudinal data on both dietary and 24-h movement behaviors due to participant burden, or cost, or difficulties in combining the 2 data sources for analyses. Although both dietary and 24-h movement behaviors are constrained to a 24-h day, how their respective data are collected and processed results in time-series data sets on different scales, making them challenging to combine for analysis. For example, accelerometer data have been typically collected in predefined epochs (i.e., movement counts in different intensities are averaged across each minute of the allocated device wear time). In contrast, dietary data from food diaries or 24-h dietary recalls varies within and between individuals by eating start time, eating duration, and eating frequency, with periods of fasting (i.e., times where no intake is recorded). Further, typically only information about eating start time is collected as part of dietary assessment, which does not allow estimation of time spent eating compared with fasting. These issues are illustrated in the study by Lin et al. [49] who used on existing accelerometer and 24-h dietary recall data collected in the NHANES 2003-2006. As there was no information on duration of eating (and is not typically collected in dietary assessment), Lin et al. [49] applied generic data smoothing, assuming a 15-min eating window, to the start of each reported eating occasion to calculate energy intake per minute. This allowed the input data for the clustering analysis to be on the same time scale as the PA data, which were recorded as vertical accelerations counts per minute. Hence, future research will need to consider measurement timescales of both dietary and 24-h movement behaviors to facilitate their joint temporal pattern analysis. Improvements in digital tools and wearable devices to assess dietary and 24-h movement behaviors has increased the feasibility of “real-time” data collection of multiple behaviors in larger scaled studies. These tools and technologies reduce participant burden and are becoming more affordable with their increasing availability. Further, consideration should be given to the infrastructure and interdisciplinary expertise required for collecting, storing, managing, and combining large data sets from different sources in the planning and design of studies on the temporal patterning of dietary and movement behaviors.

The choice of methods used for pattern analysis varied across the included studies but were often justified by the study authors for their suitability to answer the research question and appropriateness for the data type (e.g., continuous, categorical, or mixed) and structure (e.g., cross-sectional, longitudinal, multilevel, and multidimensional). For example, group-based trajectory modeling approaches were suited to examining long-term temporal patterns [47,48,56,57], and clustering data mining techniques (based on different algorithms) were used to examine a large number of input variables and correlates [49,50,51,54]. In some cases, >1 technique was required for data reduction purposes [49,50,54]. For example, Farrahi et al. [54] had to first simplify the temporal patterns identified by Niemelä et al. [51] to make the data suitable for the analysis of correlates using decision trees and identified 54 subgroups of active and inactive adults based on a large range of sociodemographic, behavioral, and contextual factors. The findings from studies using data mining methods may be applied to develop personalized behavioral support using mobile and wearable technology or generate empirical hypotheses for examination in future studies. Nonetheless, the selection of variables (predictors, outcomes, covariates) for inclusion in the analysis and interpretation of the resultant patterns should be guided by evidence-based theory. Further, model-based clustering approaches may provide some advantages over data mining clustering approaches because the selection of the optimal number of patterns is less subjective and is guided by probabilities and goodness-of-fit indicators estimated from statistical models.

The novel clustering method used by Yang et al. [57] simultaneously considered both predictors and MVPA in the model-based clustering procedure and allowed them to identify important contextual predictors of higher MVPA unique to groups with differing MVPA temporal patterns. Such information may be useful for developing interventions tailored to subpopulation’s specific needs regarding appropriate social/family support mechanisms or changes to the home, school, or neighborhood environment. Despite the potential utility of these methods for examining the temporal patterning of dietary and 24-h movement behaviors, and their accompanying contextual factors, the types of patterns examined varied in their level of detail (e.g., day-to-day overall time compared with minute-by-minute bouts of time spent in different movement intensities) and the methods and the statistical analyses involved a complex set of steps, were described using field-specific and/or statistical jargon, or were conducted using specialized/custom software packages. Clear documentation of novel methods, in particular the statistical criteria used to determine the patterns, and open-source code analysis tools may facilitate the application and refinement of such methods in future studies [69]. Further, development of a framework to standardize the characterization (i.e., terminology, definitions) and reporting of temporal behavior patterns and multidisciplinary collaborations across the fields of nutrition, PA, sleep, statistics, computer science, and computer engineering may further advance this field of research. In fact, a framework for defining and reporting waking behavior patterns has recently been published, with the aim to progress the synthesis of the study findings in this field [9].

As described earlier, the methods used in the included articles were highly technical, and so data visualization methods were often used as an adjunctive tool for conceptualizing the temporal patterns. Dietary and 24-h movement behaviors are compositional in nature, meaning that time in one behavior will affect time in another behavior in each 24-h day [70,71]. Hence, visualization strategies may be useful for mapping behaviors by population subgroups across times of the day, geographical spaces, and environments. For example, Zhao et al. [53] presented estimated means for time spent in different 24-h movement behaviors across the day (i.e., behavioral compositions) using novel spatial and data visualization techniques to facilitate understanding of PA, SED, and sleep temporal patterns in youth and the contexts in which they occur. Further, the authors proposed such visualization techniques could add value to studies using compositional data analysis whereby time use differences between groups are presented as isometric log ratio coordinates, which may be challenging to interpret on their own. Jaeger et al. [59] visually mapped categories of foods consumed at eating occasions across time of day and contexts such as location of eating and activities while eating to provide insights into the meal formats (i.e., combination and sequencing of food intake) and the social and cultural practices involved in daily eating routines. However, given the large variation in food choices across eating occasions and contexts, collecting and coding the dietary data to capture this complexity involves substantial participant and researcher burden; integrating more nuanced contextual data with automatically coded 24-h recall data may be a more feasible way to progress research on daily eating routines and their contextual influences.

A strength of this review included a comprehensive search strategy, focusing on methods to analyze the temporal patterning of 4 key health behaviors (diet, PA, SED, and sleep) and their contextual correlates. However, the literature search for this review was last updated in 2022, and we did not include studies of non-English language, gray literature, or conference proceedings. Thus, we expect more articles to have since been published, given this is a rapidly evolving area of research. Of note, only 1 new analytic approach (e.g., compositional data analysis) was identified in the 6 studies included from the updated search. This review therefore provides important insights into the analytic methods available to researchers for examining the temporal patterning of dietary and 24-h movement behaviors.

In conclusion, this scoping review identified a range of methods that have been used to analyze the temporal patterning of dietary and movement behaviors, as well as their contextual correlates. Although the studies included in this review were diverse in terms of their analytic methods, behavior input variables, and periods examined, the evidence to date suggests temporal patterns characterized by high SED and low PA are associated with poorer health outcomes, than those with low SED and high PA. However, further research is needed to elucidate the importance of context in which high SED and PA occurs, considering existing evidence to suggest leisure time PA is more beneficial to health than occupational PA [72]. Further, standardizing the reporting of methods used to derive patterns and permission to use and edit open-source code may facilitate the application and continued development of new methods.

The ability of identified methods to incorporate contextual information about the temporal patterns yielded insights into the role of social and environmental factors in promoting temporal behavior patterns. Future research examining existing microlongitudinal data or using study designs with daily assessments of behavioral and contextual factors may help elucidate the health implications of different temporal patterns and their determinants. However, this may require advancing data collection through use of wearables, smartphone, and geographic information system technology and the use of existing data on weather, green spaces and parks, food environments, and local infrastructure. Finally, this review identified only 1 study on the temporal patterning of dietary and 24-h movement behaviors, suggesting further research is needed to develop methods for analyzing dietary behaviors together with 24-h movement behaviors across the intensity spectrum. Overall, progression of the methods and procedures used to collect, integrate, and analyze dietary, movement and contextual data and multidisciplinary collaboration among nutrition, PA and sleep researchers, statisticians, and computer scientists/engineers will be important for advancing our understanding of temporal behavior patterns in relation to health and optimizing future behavioral and public health interventions.

## Author contributions

The authors’ responsibilities were as follows – all authors: conceptualized the review and had intellectual input into the review protocol; SEC: conducted the database searching and article retrieval; RML, SEC: screened the articles and conducted the data extraction; RML: critically analyzed the studies within the review and drafted the manuscript; SAM: supervised the study; and all authors: read and approved the final manuscript.

## Funding

Seed funding from the Institute of Physical Activity and Nutrition, Deakin University, was used to support this review. RML is supported by a National Health and Medical Research Council Emerging Leadership Fellowship (APP1175250). NDR was supported by a National Heart Foundation of Australia Future Leader Fellowship (ID 101895). The sources of funding had no role in the review design, article screening processes, analytic synthesis and interpretation of findings, or writing of the manuscript. There are no restrictions regarding the publication of this review.

## Conflict of interest

HAE-M (Heather A Eicher-Miller) is on the editorial board of *Advances in Nutrition* and played no role in the Journal’s evaluation of the manuscript. RML, SEC, NDR, RM, and SAM declare that they have no competing interests.
