# Supplementary material for: Analytic Methods for Understanding the Temporal Patterning of Dietary and 24-H Movement Behaviors: A Scoping Review
Source: Adv Nutr. 2024 Jul 18;15(8):100275. doi: 10.1016/j.advnut.2024.100275 (PMC11347858; doi:10.1016/j.advnut.2024.100275)
Supplement: Multimedia component 1 [file mmc1.docx]

**Supplementary File 1.** Key search terms identified under each main concept of the review and example search strings for each database

| **Concept** | **Search Strings** |
| --- | --- |
| 1. Behaviours | "diet*" OR "food intake" OR “eating” OR “meal*” OR “snack*” OR “sleep*” OR “sedentary behav*” OR “sedentary time” OR “screen time” OR "physical* activ*" OR "exercise*" OR “acceleromet*” OR “activity track*” OR “lifelogging” OR “heart rate” OR “inclinomet*” OR “GPS” OR “Bluetooth proximity” OR “device-based” |
| 1. Behaviour context | “ecological momentary assessment” OR “wearable camera*” OR “environment*” OR “context*” OR “situation*” OR “location*” OR “domain*” |
| 1. Temporal patterning | “temporal pattern*” OR “tim*” OR “time-use” OR “cluster*” OR “profil*” OR “longitudinal” OR “trajectories” OR “phenotypes” OR “typologies” OR “pattern*” OR “temporal association*” OR “fragmentation” |
| 1. Novel statistical methods | “pattern recognition” OR “algorithm*” OR “artificial intelligence” OR “machine learning” OR “deep learning” OR “data mining" OR “composition*” OR “CoDA” OR “cluster*” OR “class” OR “factor*” OR “multilevel analysis” OR “multilevel model*” OR “time-series” OR “time sequenc*” OR “signal alignment” OR “dynamic time warping” |

**Example search strings for each database**

1. **Embase**

**('diet*':ti,ab,kw OR 'food intake':ti,ab,kw OR 'eating':ti,ab,kw OR 'meal*':ti,ab,kw OR 'snack*':ti,ab,kw OR 'sleep*':ti,ab,kw OR 'sedentary behav*':ti,ab,kw OR 'sedentary time':ti,ab,kw OR 'screen time':ti,ab,kw OR 'physical* activ*':ti,ab,kw OR 'exercise*':ti,ab,kw OR 'acceleromet*':ti,ab,kw OR 'activity track*':ti,ab,kw OR 'lifelogging':ti,ab,kw OR 'heart rate':ti,ab,kw OR 'inclinomet*':ti,ab,kw OR 'gps':ti,ab,kw OR 'bluetooth proximity':ti,ab,kw OR 'device-based':ti,ab,kw) AND ('ecological momentary assessment':ti,ab,kw OR 'wearable camera*':ti,ab,kw OR 'environment*':ti,ab,kw OR 'context*':ti,ab,kw OR 'situation*':ti,ab,kw OR 'location*':ti,ab,kw OR 'domain*':ti,ab,kw) AND ('temporal pattern*':ti,ab,kw OR 'tim*':ti,ab,kw OR 'time-use':ti,ab,kw OR 'cluster*':ti,ab,kw OR 'profil*':ti,ab,kw OR 'longitudinal':ti,ab,kw OR 'trajectories':ti,ab,kw OR 'phenotypes':ti,ab,kw OR 'typologies':ti,ab,kw OR 'pattern*':ti,ab,kw OR 'temporal association*':ti,ab,kw OR 'fragmentation':ti,ab,kw) AND ('pattern recognition':ti,ab,kw OR 'algorithm*':ti,ab,kw OR 'artificial intelligence':ti,ab,kw OR 'machine learning':ti,ab,kw OR 'deep learning':ti,ab,kw OR 'data mining':ti,ab,kw OR 'composition*':ti,ab,kw OR 'coda':ti,ab,kw OR 'cluster*':ti,ab,kw OR 'class':ti,ab,kw OR 'factor*':ti,ab,kw OR 'multilevel analysis':ti,ab,kw OR 'multilevel model*':ti,ab,kw OR 'time-series':ti,ab,kw OR 'time sequenc*':ti,ab,kw OR 'signal alignment':ti,ab,kw OR 'dynamic time warping':ti,ab,kw) AND [1997-2020]/py**

**AND 'human'/de AND ('Article'/it OR 'Conference Paper'/it)**

1. **Ebsco**

( "diet*" OR "food intake" OR “eating” OR “meal*” OR “snack*” OR “sleep*” OR “sedentary behav*” OR “sedentary time” OR “screen time” OR "physical* activ*" OR "exercise*" OR “acceleromet*” OR “activity track*” OR “lifelogging” OR “heart rate” OR “inclinomet*” OR “GPS” OR “Bluetooth proximity” OR “device-based” ) AND ( “ecological momentary assessment” OR “wearable camera*” OR “environment*” OR “context*” OR “situation*” OR “location*” OR “domain*”) ) AND ( “temporal pattern*” OR “tim*” OR “time-use” OR “cluster*” OR “profil*” OR “longitudinal” OR “trajectories” OR “phenotypes” OR “typologies” OR “pattern*” OR “temporal association*” OR “fragmentation” ) AND ( “pattern recognition” OR “algorithm*” OR “artificial intelligence” OR “machine learning” OR “deep learning” OR “data mining" OR “composition*” OR “CoDA” OR “cluster*” OR “class” OR “factor*” OR “multilevel analysis” OR “multilevel model*” OR “time-series” OR “time sequenc*” OR “signal alignment” OR “dynamic time warping” )

1997-2020, Human, Journal article

1. **ProQuest**

• NOT (animals AND animal behavior AND rodents AND fish)

• NOT (Review AND Commentary AND Editorial AND Literature Review AND News AND Correspondence AND Letter To The Editor AND Correction/Retraction AND Front Page/Cover Story AND Interview AND Market Research AND Speech/Lecture)

• • • • NOT (Nursing & Allied Health Database AND Biological Science Database AND Environmental Science Database)

Date: After 31 December 1996

Source type

Conference Papers & Proceedings, Scholarly Journals

Language

English

**Supplementary File 2. Scoping Review inclusion criteria**

| *Participants* | Humans. Studies on non-human subjects will be excluded    No age or health status restrictions will be applied |
| --- | --- |
| *Concept(s)* | The overarching concept for this scoping review are to identify contemporary methods for examining the co-patterning of diet and activity behaviours, including the contexts in which they occur. Studies should include the following concepts:   - At least two diet and/or activity behaviours (diet, sleep, SED, PA) OR at least one of the diet/activity behaviours together with information about the respective behaviour context. Patterning of multiple dimensions of the same behaviour category may be included (e.g., bouts and breaks of SED, LPA and MVPA, sleep quality and quantity, meal frequency and meal content). - A temporal assessment of diet/activity behaviours with no limit to the duration of the time segments examined. For example, we are interested in studies that assess behaviours/context across episodes, occasions, epochs, hours, days, months, years etc. - A non-contemporary and multivariate analytic approach to determine co-patterns (i.e., data mining techniques, composition analysis, latent class/profile analysis). Studies will be excluded if they only used conventional multivariate statistical methods (i.e., linear/logistic regression models or generalised estimating equations) - Examines diet and activity behaviour, including context, over time as an integrated and multidimensional pattern (e.g., bouts of PA and breaks of SED as part of a daily time composition; frequency and timing of all meals across a day captured together [i.e., not analysed separately in models]. Studies will be excluded if they only examine temporal or time-specific associations (e.g., a context variable predicting daily total of an activity variable using GLM or MLMs) |
| *Context(s)* | No restrictions to study settings or context will be applied |
| *Types of evidence sources* | Journal articles or full conference papers published from Jan 1, 1997  English language only; studies written in languages other than English will be excluded  Quantitative studies with the following experimental and epidemiological study designs will be considered:   - Cross-sectional - Longitudinal   - Prospective and retrospective (including nested) cohort studies   - Randomized and non-randomized controlled trials   Qualitative studies, reviews, and conference abstracts will be excluded |

**Supplementary File 3: A description of the analytic approaches used to examine temporal behaviour patterns in the included studies**

| **Statistical approach; Author (year)** | **Details of approach** | **Software used** | **Measures of robustness** | **Strengths** | **Limitations** |
| --- | --- | --- | --- | --- | --- |
| Canonical correspondence analysis (CCA)  Jaegar (2009) | CCA is a form of correspondence analysis that relates one set of categories to another set of categories. The analysis determines which category values are predicted by the contextual variables and visualises these associations on the correspondence map. | NR | Significance of relationships between the sets of input variables and scree plot of eigen values | Reveals relationships between sets of categorical variables which can be visualised on a map. Can superimpose dimensional correspondence maps to allow more in-depth exploration of patterns. | Non convergence when too many binary variables have a zero value (original food categories had to be condensed. Exploratory - does not support significance testing. Variable centred and doesn't classify individual into groups. |
| Clustering (Machine Learning [ML] methods)  Ferrahi (2020)  Niemäla (2019) | Decision trees with X-means clustering algorithm. X-means is an expansion of the K-means algorithm and forms groups in which the intra-cluster homogeneity is high and inter-cluster homogeneity is low. | Weka, version 3.8.1 | NR | Can be efficiently applied to large amounts of continuous accelerometer-measured activity intensities over consecutive days. Practical when the number of clusters is not known a priori. | NR |
| Clustering (ML methods)  Ferrahi (2020) | Decision trees with CHAID algorithm. Partition the data based on predictor variables to form homogenous subgroups with respect to the outcome variable. Predictors in the higher layers of tree hierarchy are more important predictors. | IBM SPSS Statistics for Windows, version 25.0 | 10-fold cross validation was used to create and validate the model. The confusion matrix was used to show the proportion of participants with each outcome variable that was correctly/incorrectly classified. | CHAID can handle mixed data types and is designed to include continuous, ordinal and categorical predictors. Misclassification ok with up to 20% missing data. | Poor performance and generalizability when outcome variables are imbalanced with class overlap. Best minimized with near-balance class distributions. Misclassification increases when missing values >20% |
| Clustering (ML methods)  Lin (2022) | Distance-based  clustering analysis with dynamic time warping to determine pairwise distances between participants from multidimensional time series data. Kernel k-means, spectral clustering and hierarchical agglomerative clustering were then used to partition participants into clusters. | Python | The Sakoe-Chiba band was used to constrain the maximum temporal difference between matched entries to avoid pathological warping (i.e., matching morning activities with evening activities). Patterns were based on the Silhouhette and Dunn Index and model fit of associations with health outcomes (higher R2 or lower AIC values). | Comprehensive clustering approach that can determine joint temporal patterns from multidimensional time series dietary and PA data. Was able to combine two different data types collected on different timescales. | Requires multiple steps involving complex statistical procedures. Patterns not generalisable to other populations. |
| Clustering (ML methods)  Riou (2015) | Partitioning around medoïds (PAM) algorithm with Manhattan distance | *R*2.15.3, using the *clusterCons* and *ggplot2* packages | Resampling-based method and cluster-robustness approach called consensus clustering. Sensitivity analysis done using Euclidian distance, k-means and hierarchical clustering approaches | Robust approach to clustering through consensus with other clustering methods | Number of optimal clusters and interpretation of clusters varied type of clustering method |
| Clustering (novel model-based method)  Yang (2022) | Clustering using a novel one-step procedure which simultaneously conducts clustering and predictor variable selection using linear mixed-effect models with shrinkage penalties on the fixed and random effects | *R,* using *cluster-splmm* package | Data was firstly standardised and minimum BIC values determined the optimal cluster number. Final model gave best cluster number, goodness of fit and parsimony | Clustering is suitable for high-dimensional longitudinal and multilevel data.  All variables are considered simultaneously for selection into the model.  Within cluster variation was small and showed good classification accuracy | Some important predictor variables may be overlooked when there is small within-cluster variation but large difference between-clusters. |
| Clustering (model-based latent profile analysis)  Vidal Bustamante (2022) | Latent profile analysis is a finite mixture model that assigns participants into latent clusters based on observed continuous data. Participants are classified into clusters based on membership probabilities estimated from the finite mixture model. | R *mclust* package v5.4.7 | Model fit was indicated by a range of goodness of fit measures (i.e., BIC). Clusters were externally validated by examining correlations with independently collected data (academic performance and psychological stress) | Cluster membership is based on probabilities estimated from statistical models and is less arbitrary than other clustering approaches.  The *mclust* package offers a dimensionality reduction method that can be used to visualise and examine maximal differences between the clusters, and in relation to the original variables | Approach assumed a multivariate Gaussian distribution, and cluster membership may be affected by missing data. Sample was small student group and limited generalisability and the number of clusters that could be determined |
| Clustering  (model-based trajectory analysis)  Farooq (2021a)  Farooq (2021b) | Group-based multi-trajectory analysis estimates trajectories for two or more variables over time and connects them via conditional probabilities | STATA v14.0 (TX: Statacorp LP) plugin | Model fit indicated by smallest BIC and models with outlier groups (<2%) not considered. Average posterior probabilities for group membership >0.7 indicated model adequacy | Can identify joint trajectories of behaviour patterns over three or more timepoints. Trajectories can then be further analysed with predictors/outcomes using conventional approaches (e.g., ANOVA, regression models etc) | Limited generalisability, missing data/loss to follow up may bias estimates. Need large enough samples to stratify analysis for key confounders such as sex or SES |
| Clustering  (model-based trajectory analysis)  Parker (2021) | Parallel process  growth mixture models with regressions among the random  effects were used to identify shared categorical latent classes for both MVPA and SED processes | Mplus, version 8 | Mixture models were adjusted for school-level and age difference. Seven models were examined with lower AIC and BIC, and interpretability (latent class size) indicating better model fit | Can identify distinct latent groups based on parallel trajectories of two behaviours over time. Can adjust for confounding variables and handle missing data between time points | Limited generalisability and data-driven nature meant that no obvious “unhealthiest” reference group was observed, which made subsequent analyses with correlates challenging |
| Compositional data analysis (CoDA)  Gába (2021) | CoDA maps the compositional data to the real space by transforming the absolute values in the composition into sets of log-ratios which includes all relative information about one part given the remaining parts of the 24h composition | R Statistical Software, version 4.0.2 (R Foundation for Statistical Computing, Vienna, Austria) using the robCompositions package. | Zeros were replaced using the Bayesian-multiplicative method and robust estimators were used to minimise influence of any outlying values | Can be used to examine time spent in context specific behaviours across a 24 h day and the theoretical effect of time reallocations from unhealthy to healthy behaviours in compositional isotemporal substitution models. Addresses the collinear and compositional nature of PA and SED data. | Need larger samples to do subgroup analyses and include multiple predictors and confounders in compositional regression models. Omitting sleep from analysis may lead to biased estimates. Zero values may be difficult to handle since CoDA uses log transformed data. |
| Factor analysis and latent growth-curve models (LCGM)  Wang (2021) | Factor analysis was used to determine daily PA patterns. Following this, LGCMs with random/fixed effects were fitted to the daily PA panel data. LGCMs are hierarchical linear models that account for within-subject and between-subject variations. | Stata V15.2 | Four sets of ancillary models were used to check for evidence of a quadratic or piecewise trend. Backward elimination strategies (removing one variable at a time) and its impact on the model specifications (AIC, BIC, RMSEA and CFI values) were assessed. | The LCGM can handle multilevel, multiscale data and could examine multiple factors at once. | Doesn't account for endogeneity bias. For example, daily PA level might affect sleeping minutes and another set of LCGMs need to be estimated to address this any reverse effects |
| Principal Components Analysis (PCA) and clustering (ML methods)  Mesquita (2017) | PCA (set to capture minimum of 60% variance) analysis initially reduced the data to 3 principal components. K-means cluster analysis was then applied to the 3 components. | Matlab R2012b (Mathsworks Inc., Natick, MA, USA) | CA algorithm selected was most stable under small perturbations of the dataset and normalised mean over pairwise clustering distances was used as instability measures. Cluster number was automatically selected by the program (not researchers) | Identify subgroups with distinct patterns using detailed PA measures. Shows when people spend time in different intensities during the day | Clusters were not validated, and substantial data reduction was needed (via PCA) before the CA |
| Data visualisation methods  Zhao (2019) | Ring maps and time-activity diagrams are data-driven methods for visualising space, time, and activity in association with their contextual settings. The ringmap shows spatial and temporal activity patterns over 24h. Time-activity diagrams visualise time-use patterns (timing and sequencing of activities) between different subgroups | R-software open-source visualisation tools | NR | Can reveal spatial, temporal, sequential activity patterns and facilitates easy comparison between groups and within a group over time and compositional activities. Can easily be applied to data that involve space, time, and activity (i.e., time-diary data). Ideal for visualising clinical data that change over circadian time (e.g., blood pressure). | Exploratory method with limited generalisability. At the individual-level, time–activity diagram can only effectively display a few hundred individuals. For ring maps, visualisations were based on mean values and didn’t account for within-subject variability. |

AIC, Akaike Information Criterion; BIC, Bayesian Information Criterion, CCA, canonical correspondence analysis; CHAID, Chi2 Automatic Interaction Detection; CFI, Comparative Fit Index; PCA, principal components analysis; LGCM, latent growth curve models; LPA, latent profile analysis; ML, machine learning; RMSEA, Root Mean Square Error of Approximation

**Supplementary Figure 1: PRISMA 2020 flow diagram showing searches of databases and other sources**

**Identification of studies via databases and registers**

**Identification of studies via other methods**

Records identified from:

Citation searching (n = 3)

Records removed *before screening*:

Duplicate records removed (n=392)

Records removed for other reasons: (n=0)

Records identified from updated search (2021-22)*

Databases:

(n = 6485)

Records removed *before screening*:

Duplicate records removed (n=5301)

Records removed for other reasons: (no author) (n=107)

Records identified from original search*

Databases:

(n = 32607)

**Identification**

Records screened (n=6093)

Records excluded**

(n=6058)

Records excluded**

(n=27102)

Records screened (n=27199)

Reports not retrieved

(n = 0)

Studies sought for retrieval

(n = 3)

Studies excluded (n=91)

Did not analyse behaviours as an integrated temporal pattern (n=41)

No temporal assessment of diet/activity behaviours (n=24)

Insufficient diet/activity behaviours examined (n=12)

Conference abstract (n=2)

Not in English (n=1)

Review Paper (n=1)

Daily activity compositions with no temporal analysis (n=5)

Other reasons (n=5)

Studies excluded (n=29)

Did not analyse behaviours as an integrated temporal pattern (n=15)

No temporal assessment of diet/activity behaviours (n=2)

Insufficient diet/activity behaviours examined (n=4)

Other reasons (n=7)

Review paper (n=1)

Studies assessed for eligibility.

(n=35)

Studies assessed for eligibility.

(n=97)

**Screening**

Studies excluded (n=1):

Insufficient diet/activity behaviours examined

Studies assessed for eligibility

(n = 3)

Studies included in review

(n=14)

**Included**

*From:*  Page MJ, McKenzie JE, Bossuyt PM, Boutron I, Hoffmann TC, Mulrow CD, et al. The PRISMA 2020 statement: an updated guideline for reporting systematic reviews. BMJ 2021;372:n71. doi: 10.1136/bmj.n71. For more information, visit: <http://www.prisma-statement.org/>
